# Epidemiology, Histopathologic Patterns, and Clinical Characteristics of Gestational Trophoblastic Diseases in Ethiopia: A Systematic Review and Meta‐Analysis Study

**DOI:** 10.1155/ogi/6438666

**Published:** 2026-06-11

**Authors:** Abrham Tesfaye Habteyes, Fekadeselassie Teferi Mekonen, Ashebir TufaTelila

**Affiliations:** ^1^ Department of Nursing, College of Health Sciences, Ethiopian Defence University, Bishoftu, Ethiopia, ethdu.edu.et; ^2^ Department of Pharmacy, College of Health Science, Addis Ababa University, Addis Ababa, Ethiopia, aau.edu.et; ^3^ Department of Midwifery, St’ Paul Hospital Millennium Medical College, Addis Ababa, Ethiopia

**Keywords:** clinical features, gestational trophoblastic diseases, histopathology, prevalence

## Abstract

**Background:**

Gestational trophoblastic diseases (GTDs) are rare trophoblastic disorders that contribute substantially to maternal morbidity. However, the epidemiology and clinical characteristics of GTD‐related evidence from Ethiopia remain limited and scattered. Consequently, the national burden, histopathological distribution, and clinical presentation of GTD in Ethiopia are not well defined. In response to this gap in the literature, this systematic review and meta‐analysis sought to quantify the pooled prevalence of GTD and to synthesize available evidence on its histopathological and clinical profiles in Ethiopia.

**Methods:**

PubMed, Scopus, Embase, the Cochrane Library, CINAHL, and Google Scholar were systematically searched, supplemented by manual searches of local journals and institutional repositories. The study protocol was registered and implemented in accordance with the Preferred Reporting Items for Systematic Reviews and Meta‐Analyses (PRISMA) 2020 guidelines. All studies reporting the prevalence of GTD in Ethiopia up to December 5, 2025, were eligible for inclusion. To stabilize variances when pooling proportions, a random‐effects meta‐analysis was performed by using the Freeman–Tukey double arcsine transformation. Publication bias and heterogeneity across studies were evaluated.

**Results:**

Seven studies met the inclusion criteria, out of a total of 436 records collected and evaluated, comprising 121,248 women who had delivered. The pooled prevalence of GTD in Ethiopia was 0.8% (95% CI: 0.2%–1.3%). Hydatidiform mole was the most common histopathological subtype, with complete and partial moles accounting for 47.1% and 20.7% of cases, respectively. Vaginal bleeding was the most frequently reported clinical presentation (88.4%), followed by a uterus larger than gestational age (34.8%).

**Conclusions:**

This systematic review and meta‐analysis demonstrated that the prevalence of GTD in Ethiopia is slightly higher than estimates reported from other regions. Hydatidiform mole was the predominant histopathological subtype, and vaginal bleeding was the most common clinical presentation. These results highlight the necessity of enhancing early detection and diagnostic capacity, particularly at the primary healthcare level.

## 1. Introduction

Gestational trophoblastic disease (GTD) is a condition characterized by abnormal proliferation of trophoblastic cells within the uterus. GTD lesions vary in histology and can be benign or malignant. GTD encompasses lesions with varying histopathological features that may be either malignant or benign. Malignant forms comprise invasive moles, choriocarcinoma, placental‐site trophoblastic tumors, and the rare epithelioid trophoblastic tumor, whereas benign forms include complete and partial hydatidiform moles [1, 2]. Treatment options for GTD depend on the type and severity of disease and may include surgery, such as suction curettage and hysterectomy, chemotherapy, radiotherapy, and psychological support [3].

GTD has been associated with a number of risk factors, including hormonal influences, dietary factors, multiparity, ABO blood group, extremes of reproductive age, environmental exposures, lifestyle factors, a history of spontaneous abortions, and socioeconomic status [4, 5]. Prognosis is guided by clinical and laboratory parameters, including the WHO scoring system, while serum beta‐hCG levels are key for monitoring disease activity and treatment response [6]. Advances in cytogenetic and molecular diagnostics have further improved diagnosis, clarified parental origin, aided risk stratification, and personalized management [7].

GTD arises from abnormal fertilization events, and it could result in obstetric near‐miss incidents or progress to gestational trophoblastic neoplasia (GTN). If not detected early and managed appropriately, GTD can result in substantial maternal morbidity and an increased risk of mortality due to related complications [8]. There are significant regional variations in the incidence of GTD, with the highest rates reported in Taiwan (approximately 1 per 125 live births) and the lowest rates observed in the United States (0.6–1.1 per 1000 pregnancies) [9, 10]. These differences are influenced by factors such as population genetics, maternal age distribution, reproductive patterns, nutritional status, access to early pregnancy care, and variations in diagnostic and reporting practices across regions [4, 10].

In Africa, a prospective study from Egypt reported that GTN and molar pregnancy rates are 3.2 and 13.1 per 1000 live births, respectively [11]. Similarly, a study in Dakar, Senegal, found that GTN accounted for 15.7% of diagnosed cases and 12.8% of related deaths [12]. In Ethiopia, a study conducted in the southwest of the country reported a GTD prevalence of 7.2 per 1000 deliveries, of which 90.9% were molar pregnancies (77.1% complete and 22.9% partial moles); GTN was diagnosed in 9.1% of cases, with invasive mole (6.5%) being the most common subtype, followed by choriocarcinoma (2.6%) [13].

The clinical presentation of GTD has declined in developed countries, largely due to routine early‐pregnancy ultrasound screening. In contrast, patients in low‐ and middle‐income countries often present late, with a range of symptoms [9, 10]. Vaginal bleeding is the most common presentation, but other manifestations include abdominal swelling or discomfort, excessive vomiting, passage of vesicles, a uterus larger than gestational age, hyperemesis, signs of severe preeclampsia, and, rarely, hyperthyroidism [6, 9, 10, 14]. Beyond physical symptoms, GTD can have significant emotional and psychosocial impacts. Affected women may experience fear of severe illness or death and grief over gestational loss [15]. The disease and its treatment, particularly chemotherapy, may also lead to sexual dysfunction and relationship difficulties, including reduced sexual desire, lubrication problems, and changes in partner dynamics [15, 16].

These diseases cause significant health risks, especially in low‐ and middle‐income nations like Ethiopia. Regardless of the clinical significance of GTD, no literature review has been conducted, as far as the author’s search shows, to show the cumulative data regarding its magnitude, histopathology, and clinical profiles in Ethiopia. As a result, combining the available information through a systematic review and meta‐analysis can provide more accurate and thorough knowledge of GTD in the country. The aim of this systematic review and meta‐analysis was to determine the pooled prevalence, histology, and clinical characteristics of GTD in Ethiopia.

## 2. Methods

### 2.1. Design and Registration

This systematic review and meta‐analysis was conducted following the Preferred Reporting Items for Systematic Reviews and Meta‐Analyses (PRISMA) 2020 guidelines [17] (Supporting File S2). The study protocol was registered in the International Prospective Register of Systematic Reviews (PROSPERO) under the registration number CRD42024569074, available at https://www.crd.york.ac.uk/prospero/#recordDetails.

### 2.2. Eligibility Criteria

Studies were considered eligible for inclusion based on the CoCoPop framework:

❖ **Condition (Co):** Studies reporting the prevalence of GTD.
❖ **Context (Co):** Studies conducted in any region of Ethiopia.
❖ **Population (Pop):** Women who gave birth.
❖ **Study design:** Observational studies, reporting original prevalence data on GTD.
❖ **Language:** Studies published in English.
❖ **Publication date:** Articles published up to December 5, 2025.
❖ **Publication type:** Published and unpublished studies were taken into consideration.

#### 2.2.1. Exclusion Criteria

Studies were excluded if full‐texts were not accessible, did not report GTD prevalence, scored poorly on quality assessment, or were case reports/series, editorials, or conference abstracts.

### 2.3. Search Strategy

The search strategy aimed to capture both published and unpublished studies. An initial limited search of Scopus and PubMed was conducted to identify relevant articles, followed by a comprehensive electronic search of MEDLINE (via PubMed), Scopus, Embase, Cochrane Library, CINAHL, and Google Scholar. Manual searches were also performed on local journal websites, including the *Ethiopian Journal of Health Development*, *Ethiopian Journal of Health Sciences*, *Ethiopian Journal of Medical and Health Sciences*, *Ethiopian Journal of Health and Biomedical Sciences*, *Ethiopian Medical Journal*, and *Ethiopian Journal of RH*.

The search procedure was established using a combination of medical subject headings (MeSH) phrases and free‐text keywords related to “histopathology,” “gestational trophoblastic diseases,” “clinical feature,” and “Ethiopia” and included all studies published up to December 5, 2025 (Supporting File S1). Two investigators (A.T.H. and F.T.M.) independently conducted the searches. EndNote X9 and the Covidence workflow [18] platform were used to manage and organize the search results. Google Scholar was searched to identify additional relevant studies and gray literature that may not be indexed in traditional databases. To ensure feasibility and consistency, the first 200 results, sorted by relevance, were screened.

### 2.4. Study Screening/Selection

Following the search, all the collected articles were imported to Covidence for screening. Following a pilot test, the titles and abstracts were examined, and the complete texts of possibly eligible studies were evaluated against the inclusion criteria by two reviewers (A.T.H. and F.T.M.). Reasons for exclusion at the full‐text stage were documented and reported in the review. Disagreements during screening were resolved through discussion and, if necessary, consultation with a third reviewer (A.T.T).

### 2.5. Outcome Measurement

The main objective of this study was to determine the overall prevalence of GTD in Ethiopia; the outcome data were extracted from each study as both a proportion/percentage. In the meta‐analysis, the pooled prevalence was calculated by dividing the number of events (GTD) by the entire number of women who gave birth. Information on histopathological subtypes and clinical features was also collected and summarized in tables.

### 2.6. Methodological Quality

Each included study’s methodological quality was independently appraised by two reviewers (A.T.H. and F.T.M.) using the Joanna Briggs Institute (JBI) checklist for prevalence and incidence studies [19]. This tool evaluates key criteria for cross‐sectional studies, including the following: (1) appropriate sample frame, (2) proper participant recruitment, (3) adequate sample size, (4) clear description of study subjects and setting, (5) sufficient coverage in data analysis, (6) valid methods for condition identification, (7) standardized and reliable outcome measurement, (8) appropriate statistical analysis, and (9) response rate. Studies scoring ≥ 50% on these indicators were considered high quality. Any disagreements during assessment were resolved through discussion and, if necessary, consultation with a third reviewer (A.T.T.).

### 2.7. Data Extraction

Data from the final included studies were independently extracted by two authors (A.T.H. and F.T.M.) using a Microsoft Excel–based data abstraction form. Extracted information included the authors’ names, year of publication, study region, sample size, type of GTD, clinical characteristics, study setting and sampling technique, and the proportion of different histologic types and clinical features. Disagreements during data extraction were solved through discussion and, if needed, consultation with a third reviewer (A.T.T.). The level of agreement between the two extractors was assessed using Cohen’s kappa statistic, which measures agreement beyond chance.

### 2.8. Statistical Analysis

To stabilize variances when pooling proportions, a random‐effects meta‐analysis was performed using the Freeman–Tukey double arcsine transformation because the majority of included studies reported proportions close to zero due to the rarity of GTD. This transformation improves the validity of pooled estimates and corresponding confidence intervals [20, 21]. Between‐study heterogeneity was accounted for using the DerSimonian–Laird method [22]. This approach accounts for both within‐study and between‐study variability, providing a more robust summary estimate in the presence of heterogeneity across studies [23, 24]. Pooled estimates and corresponding 95% confidence intervals were back‐transformed to the proportion scale. All analyses were conducted using STATA Version 17. Heterogeneity across studies was assessed using the I^2^ statistic, with values above 75% considered indicative of high heterogeneity. To explore sources of variability, subgroup analyses were performed based on the study region and year of publication. Additionally, sensitivity analysis was performed to assess how particular studies affected the pooled values. Certainty in the body of evidence was evaluated qualitatively based on the study design, methodological quality, consistency, and heterogeneity.

### 2.9. Assessment of Publication Bias

Publication bias was evaluated statistically using Egger’s and Begg’s statistical tests as well as graphically using a funnel plot. The funnel plot result interpretation depends on the shape of the graph; if it is symmetrical, then it is interpreted to suggest the absence of publication bias. In Egger’s statistical test, a *p* value < 0.05 shows the presence of a small study effect, and if that happens, it is handled by Duval and Tweedie’s nonparametric trim and fill analysis to estimate the number and adjust missing studies.

## 3. Results

### 3.1. Study Selection

**FIGURE 1:**
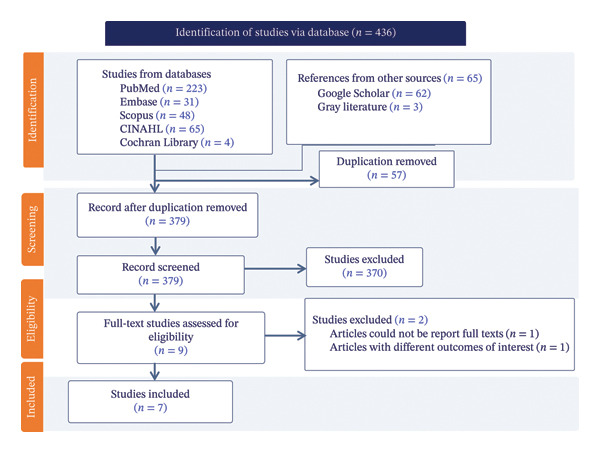
PRISMA flowchart of study selection for GTD epidemiology in Ethiopia.

The Covidence workflow software was used to handle the study selection process, which adhered to PRISMA 2020 guidelines. A total of 436 records were identified from electronic databases, including PubMed (*n* = 218), Scopus (*n* = 48), Embase (*n* = 32), CINAHL (*n* = 69), the Cochrane Library (*n* = 4), Google Scholar (*n* = 62), and gray literature sources from a local university repository (*n* = 3). After removing 57 duplicate records, 379 articles were eligible for abstract and title screening. Based on the predetermined inclusion criteria, 370 of these records were removed. Seven of the nine full‐text articles that were evaluated for eligibility fulfilled the requirements and were added to the final meta‐analysis (Figure 1).

### 3.2. Included Studies’ Baseline Characteristics

Seven studies were included in this meta‐analysis and systematic review [13, 25–30], published between 2008 and 2022. These studies were carried out throughout various Ethiopian regions, with two studies each from Addis Ababa [27, 29] and Jimma [13, 28], and one study each from Mekelle [25], Hawassa [26], and Harar [30]. Four studies employed an all‐source population (census) approach, two used systematic random sampling, and one applied multistage sampling. All included studies used cross‐sectional study design (Table 1).

**TABLE 1:** Summary characteristics of studies on GTD epidemiology in Ethiopia.

Authors	Year	Study area	Region	Case	Sample size	Prevalence	Sampling method
Negussie et al. [27]	2008	Addis Ababa	Central	93	33,438	0.30%	Census
Taye et al. [13]	2019	Jimma	Oromia	83	11,453	0.72%	Census
Yilma et al. [26]	2020	Hawassa	Sidama	194	16,957	1.20%	Census
Kahsay et al. [28]	2019	Jimma	Oromia	226	17,331	0.73%	SRS
Yibrah et al. [25]	2019	Mekelle	Tigray	45	5185	0.87%	MSS
Alemnew et al. [29]	2018	Addis Ababa	Central	124	19,683	0.63%	SRS
Barkadle et al. [30]	2022	Harar	Harar	181	17,201	1.05%	Census

*Note:* MSS: multistage sampling.

Abbreviation: SRS, systematic random sampling.

### 3.3. Methodological Quality Assessment of Included Studies

Two independent reviewers (A.T.H. and F.T.M.) examined the methodological quality of the included studies using the JBI critical appraisal checklist for observational studies [19], with scores ranging from 71.4% to 100% (Table 2). Disagreements were handled through dialog and consultation with an additional reviewer (A.T.T.). The reviewers agreed that the risk of selection, ascertainment, and nonresponse bias was low. However, all included studies scored above 50% on the quality assessment indicators and were considered high quality. Consequently, data extraction and analysis involved all the seven studies. Substantial to perfect agreement was observed between reviewers regarding study bias, with Cohen’s kappa values ranging from 0.67 to 1 (Supporting File S1). The overall certainty of evidence was considered low to moderate due to the observational nature of the evidence.

**TABLE 2:** JBI methodological quality summary of studies on the epidemiology of GTD in Ethiopia.

Study	Q1	Q2	Q3	Q4	Q5	Q6	Q7	Q8	Q9	Overall quality
Negussie et al. [27]	✓	✗	✓	✗	✓	✓	✓	✓	?	Moderate
Taye et al. [13]	✓	✓	✓	✓	✓	✓	✓	✓	✓	High
Yilma et al. [26]	✓	✓	✓	✓	✓	✓	✗	✓	✓	High
Kahsay et al. [28]	✓	✓	?	✓	✓	✓	✓	✓	✓	High
Yibrah et al. [25]	✓	✓	✓	✓	?	✓	✓	✓	✓	High
Alemnew et al. [29]	✗	?	✓	✓	✓	✓	?	?	✓	Moderate
Barkadle et al. [30]	✓	✓	✗	✓	✓	✓	✓	✗	✓	Moderate
Overall (%)	**85.7**	**71.4**	**71.4**	**85.7**	**85.7**	**100**	**71.4**	**71.4**	**85.7**	

*Note:* ✓: yes (low risk of bias), ✗: no (low risk of bias), ?: unclear. The bold values represent the proportion of included studies that fulfilled each JBI methodological quality assessment criterion.

### 3.4. Prevalence of GTDs in Ethiopia

**FIGURE 2:**
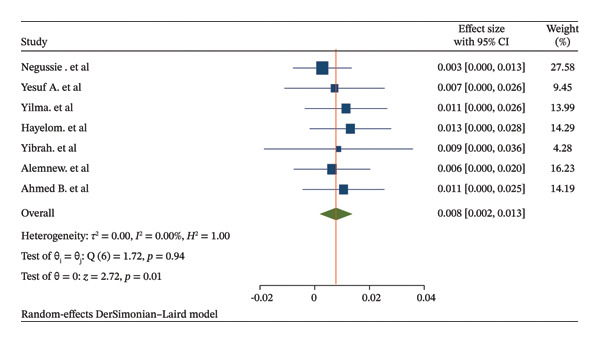
Forest plot shows the overall pooled prevalence of GTD in Ethiopia.

The highest prevalence of GTD was observed in the southern part of Ethiopia (Hawassa) at 1.20% [26], while the lowest was found in Addis Ababa at 0.30% [27]. The median prevalence across all seven studies was 0.73% (interquartile range: 0.63%–1.05%). The overall pooled prevalence of GTD in Ethiopia using a random‐effects meta‐analysis with Freeman–Tukey double arcsine transformation was 0.8% (95% CI: 0.2%–1.3%) (Figure 2). No heterogeneity was detected among the studies (*Q* = 1.72, I^2^ = 0%), indicating that the study results were consistent.

### 3.5. Subgroup Analysis

#### 3.5.1. Subgroup Analysis by Different Regions of Ethiopia

Subgroup analyses were conducted to examine regional variations in GTD prevalence across Ethiopia. The pooled prevalence ranged from 0.5% (95% CI: 0.1%–0.8%) in central Ethiopia (Addis Ababa) to 1.2% (95% CI: 1.0%–1.4%) in Sidama (Hawassa). Heterogeneity within the subgroups was low (I^2^ = 0.00%, *p* < 0.001) (Supporting File S1).

#### 3.5.2. Subgroup Analysis by Publication Year

The pooled prevalence of GTD ranged from 0.3% (95% CI: 0.3%–0.4%) in studies published in 2008 to 1.2% (95% CI: 1.0%–1.4%) in studies published in 2020. Heterogeneity within the subgroups was low (I^2^ = 0.00%, *p* < 0.001) (Supporting File S1).

#### 3.5.3. Publication Bias

**FIGURE 3:**
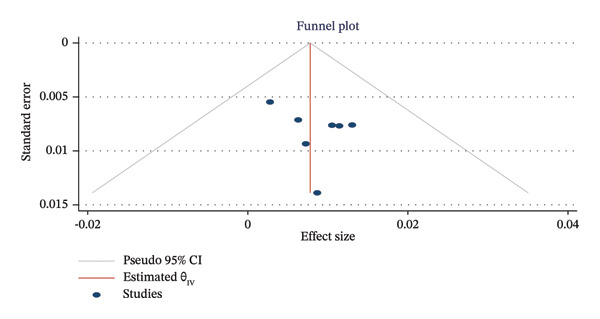
Funnel plot asymmetry for testing publication bias on studies of GTD in Ethiopia.

Egger’s regression test was not statistically significant (*B* = 1.01, SE = 1.6, *P* = 0.5273) (Supporting File S1), and visual inspection of the funnel plot revealed no asymmetry (Figure 3), suggesting that publication bias is unlikely to have affected the pooled estimates.

#### 3.5.4. Sensitivity Analysis

The sensitivity analysis was carried out successively by deleting each study to assess its impact on the overall prevalence estimate. This leave‐one‐out approach revealed that the pooled prevalence of GTD remained stable, ranging from 0.7% to 0.9%, indicating that the results were not driven by any single study (Table 3), and the forest plot is demonstrated in supporting information (Supporting File S1).

**TABLE 3:** Leave‐one‐out sensitivity analysis of pooled prevalence.

Excluded study	Prevalence (%)	95% CI	*p* value
Negussie et al. [27]	0.9	0.7%–1.0%	< 0.001
Taye et al. [13]	0.8	0.5%–1.1%	< 0.001
Yilma et al. [26]	0.7	0.5%–1.1%	< 0.001
Kahsay et al. [28]	0.8	0.5%–1.1%	< 0.001
Yibrah et al. [25]	0.8	0.5%–1.1%	< 0.001
Alemnew et al. [29]	0.8	0.5%–1.1%	< 0.001
Barkadle et al. [30]	0.7	0.5%–1.0%	< 0.001

#### 3.5.5. Histopathologic Patterns and Clinical Characteristics of GTD in Ethiopia

The histopathological patterns and clinical features of GTD in Ethiopia were examined using both a narrative synthesis and pooled prevalence analysis [13, 25–30].

#### 3.5.6. Histopathologic Patterns of GTD in Ethiopia

Six studies reported histopathological examination of surgical specimens, though some inconsistencies in reporting were noted, particularly in studies from Addis Ababa [27] and Harar [30]. Hydatidiform mole was the most common histologic type of GTD. The highest proportions of complete and partial moles were observed in Jimma (70.1% and 35.5%, respectively) [13, 28], while the lowest complete mole prevalence was reported in Mekelle (20%) [25] and the lowest partial mole in Harar (3.8%) [30]. Invasive mole and choriocarcinoma were less frequent, with the highest proportions reported in Addis Ababa (15.1% and 12.9%, respectively) [27] and the lowest in Hawassa (4.5%) [26] and Jimma (2.6%) [13] (Table 4).

**TABLE 4:** Histopathologic patterns of GTD in Ethiopia.

Author, ref	Complete mole (%)	Partial mole (%)	Invasive mole (%)	Choriocarcinoma (%)
Taye et al. [13]	70.1	20.8	6.5	2.6
Yilma et al. [26]	68.2	21.9	4.5	5.4
Kahsay et al. [28]	48.7	35.0	8.4	4.9
Yibrah et al. [25]	20	22.2	6.7	2.2
Barkadle et al. [30]	28.7	3.8	^∗^	6.0
Negussie et al. [27]	^∗^	^∗^	15.1	12.9

^∗^Represents a histopathologic finding that was not reported in the articles.

The pooled prevalence of GTD histologic subtypes showed that hydatidiform mole was the most common, with complete moles accounting for 47.1% (95% CI: 25.2%–69.1%) and partial moles for 20.7% (95% CI: 14.6%–26.9%). Invasive mole and choriocarcinoma were less frequent, with pooled prevalence of 8.2% (95% CI: 4.7%–11.8%) and 5.7% (95% CI: 2.0%–9.3%), respectively (Supporting File S1).

#### 3.5.7. Clinical Characteristics of GTD in Ethiopia

Four studies reported the clinical features of GTD, although there were some inconsistencies in reporting. Vaginal bleeding was the most common presenting symptom, occurring in 82.2%–100% of cases, followed by a large‐for‐gestational‐age uterus, reported in 3.6%–66.8% of patients. Passage of vesicles was observed in 2.4%–30.4% of cases. Thyrotoxicosis was reported in 32.5% of patients in Jimma [13], and preeclampsia occurred in 22.2% of patients in Mekelle [25] (Table 5).

**TABLE 5:** Clinical characteristics of gestational trophoblastic disease in Ethiopia.

Clinical features	Yesuf A. et al.	Yibrah B. et al.	Yilma M. et al.	Ahmed B. et al.
Vaginal bleeding	84.5%	82.2%	87.1%	100%
Passage of vesicles	2.4%	20.0%	30.4%	^∗^
Big for date uterus	3.6%	31.1%	37.6%	66.8%
Vomiting	16.9%	26.7%	^∗^	^∗^
Hyperthyroidism	32.5%	17.8%	^∗^	^∗^
Preeclampsia	14.4%	22.2%	^∗^	^∗^

^∗^Represents a symptom that was not reported in the articles.

The pooled analysis of clinical features among GTD patients showed that vaginal bleeding was the most common symptom, affecting 88.4% (95% CI: 83.8%–93.1%), followed by a large‐for‐gestational‐age uterus in 34.8% (95% CI: 10.5%–59.0%). Hyperthyroidism and vomiting were reported in 25.2% (95% CI: 10.7%–39.6%) and 21.8% (95% CI: 12.2%–31.4%) of patients, respectively. Passage of vesicles occurred in 17.6% (95% CI: 0.3%–34.9%), and preeclampsia in 18.3% (95% CI: 10.7%–25.9%) (Supporting File S1).

## 4. Discussion

The main aim of conducting this meta‐analysis study was to provide a comprehensive estimate of the prevalence, histopathological patterns, and clinical features of GTD in Ethiopia. To our knowledge, this is the first study to synthesize national‐level data on GTD, offering a clearer understanding of its burden across different regions. The pooled prevalence of 0.8% highlights that GTD remains an important cause of maternal morbidity in Ethiopia, with significant variations in histopathology and clinical presentation that have implications for early diagnosis and management strategies at the national level.

The results of this meta‐analysis indicate that the overall pooled prevalence of GTD in Ethiopia was 0.8% (95% CI: 0.2%–1.3%). This finding is consistent with a study from Nigeria, which also reported a prevalence of 0.8% [31]. However, the pooled prevalence in Ethiopia is higher than reports from several other countries, including Saudi Arabia (0.13%) [32], India and England (0.14%) [33, 34], Italy and Bhutan (0.2%) [35, 36], India (0.26% and 0.42%) [37, 38], Oman (0.3%) [39], Iraq (0.32%) [40], and Pakistan (0.41%) [41]. Conversely, it is lower than prevalence estimates reported in other African countries, such as Nigeria (4.6%) and Tanzania (21%) [42, 43].

The observed differences in GTD prevalence between countries may reflect variations in diagnostic criteria and reporting practices. In settings with limited access to ultrasound and pathology services, molar pregnancies may go undetected or be misclassified as spontaneous abortions [44]. Nutritional factors, such as deficiencies in carotene and folic acid, may also contribute to higher rates in some populations [45]. Additionally, differences in study design and sample size could influence prevalence estimates; for example, the study in Tanzania included only 200 deliveries, and most of the comparator studies were single‐center primary studies, which may limit their generalizability.

This review demonstrated that hydatidiform mole was the predominant histologic type of GTD, accounting for 47.1% of cases for complete mole and 20.7% for partial mole, followed by invasive mole (8.2%) and choriocarcinoma (5.7%). These findings are consistent with reports from other countries [31, 35, 36, 38, 43]. The higher proportion of hydatidiform moles may be explained by the fact that they represent the initial stage in the spectrum of GTD and occur more frequently than malignant forms. Additionally, hydatidiform moles are often detected during the evaluation of early pregnancy loss, which likely contributes to their higher reported prevalence compared with invasive mole and choriocarcinoma [46].

Regarding clinical presentation, this meta‐analysis found that vaginal bleeding was the most common feature of GTD, occurring in 88.4% of cases, followed by a large‐for‐gestational‐age uterus (34.8%), hyperthyroidism (25.2%), and vomiting (21.8%). Passage of vesicles was reported in 17.6% of patients, and preeclampsia in 18.3%. These findings are consistent with previous studies [33, 40, 41, 43]. The predominance of vaginal bleeding is likely due to abnormal trophoblastic proliferation and degeneration of chorionic villi, which disrupt uterine blood vessels and cause detachment from the endometrium, making bleeding the earliest noticeable sign of GTD [46]. Uterine enlargement, or a large‐for‐gestational‐age uterus, results from excessive trophoblastic proliferation and villous edema, particularly when the disease is not diagnosed early [47].

Despite providing valuable insights, this systematic review and meta‐analysis acknowledged some limitations. First, all included studies employed cross‐sectional designs, which are susceptible to residual confounding and may lead to over‐ or underestimation of prevalence estimates. Second, not all regions of Ethiopia were represented due to the limited number of available studies, which may affect the national generalizability of the findings. Third, the final analysis included a small number of studies, and some had relatively small sample sizes. These limitations highlight the need for further research in underrepresented regions to improve national representativeness, fill evidence gaps, and strengthen the overall evidence base on GTD in Ethiopia.

## 5. Conclusion

This meta‐analysis demonstrated that the overall pooled prevalence of GTD in Ethiopia is slightly higher than estimates reported from other regions. Hydatidiform mole was identified as the predominant histologic type, and vaginal bleeding was the most common clinical feature. These findings highlight the importance of strengthening early detection and diagnostic capacity, particularly at the primary care level, through community education and integration of GTD awareness into routine prenatal care. The high prevalence of vaginal bleeding and hydatidiform moles underscores the need for training healthcare providers to recognize early symptoms. Future research should explore factors contributing to the slightly elevated prevalence in Ethiopia and prioritize prospective cohort studies to better understand disease progression and outcomes.

## 6. Implications of the Study

This study provides the first comprehensive national estimate of the prevalence, histopathological patterns, and clinical features of GTD in Ethiopia to fill a critical knowledge gap and offers a robust foundation for public health actions. The findings underscore the need to strengthen early detection and diagnostic capacity at the primary care level. Integrating GTD awareness into routine prenatal care and community education programs can improve timely diagnosis and management. Additionally, the study identifies gaps in limited research on the field, emphasizing the importance of future prospective studies to better understand risk factors, disease progression, and outcomes, which can inform evidence‐based public health strategies and maternal health policies.

**Nomenclature**
GTDs: Gestational trophoblastic diseases
GTN: Gestational trophoblastic neoplasia
JBI: Joanna Briggs Institute
PSTTs: Placental‐site trophoblastic tumors
PROSPERO: International Prospective Register of Systematic Reviews
PRISMA: Preferred Reporting Items for Systematic Reviews and Meta‐Analyses
WHO: World Health Organization

## Author Contributions

Abrham Tesfaye Habteyes and Ashebir TufaTelila developed the protocol and were involved in the design, selection of the study, data extraction, statistical analysis, and development of the initial drafts of the manuscript. Abrham Tesfaye Habteyes, Fekadeselassie Teferi Mekonen, and Ashebir TufaTelila were involved in data extraction, quality assessment, statistical analysis, and revision. Ashebir TufaTelila also proofread and edited the manuscript thoroughly. Abrham Tesfaye Habteyes and Ashebir TufaTelila prepared the final draft of the manuscript.

## Funding

No funding was received for this manuscript.

## Disclosure

All authors read and approved the final draft of the manuscript.

## Ethics Statement

The authors have nothing to report.

## Consent

The authors have nothing to report.

## Conflicts of Interest

The authors declare no conflicts of interest.

## Supporting Information

Additional supporting information can be found online in the Supporting Information section.

## Supporting information


**Supporting Information 1** Supporting File S1: Contains the JBI critical appraisal checklist for included studies, summary of quality assessment, detailed search strategies, regression‐based Egger test for small‐study effects, subgroup analysis forest plot outputs, pooled prevalence of different histologic types of GTD in Ethiopia, pooled proportion of various clinical features among GTD patients in Ethiopia, sensitivity analysis forest plot outputs, and pooled prevalence of GTD after trim‐and‐fill analysis.

**Supporting Information 2** Supporting File S2: PRISMA 2020 checklist.

## Data Availability

On reasonable request, the corresponding author (Abrham Tesfaye, abrhamtesfaye95@gmail.com) makes available the generated and/or analyzed datasets.
