# Supplementary material for: Epidemiology, Histopathologic Patterns, and Clinical Characteristics of Gestational Trophoblastic Diseases in Ethiopia: A Systematic Review and Meta‐Analysis Study
Source: Obstet Gynecol Int. 2026 Jun 11;2026:6438666. doi: 10.1155/ogi/6438666 (PMC13254813; doi:10.1155/ogi/6438666)
Supplement: Supplementary file 1 — Supporting Information 1 Supporting File S1: Contains the JBI critical appraisal checklist for included studies, summary of quality assessment, detailed search strategies, regression‐based Egger test for small‐study effects, subgroup analysis forest plot outputs, pooled prevalence of different histologic types of GTD in Ethiopia, pooled proportion of various clinical features among GTD patients in Ethiopia, sensitivity analysis forest plot outputs, and pooled prevalence of GTD after trim‐and‐fill analysis. [file OGI-2026-6438666-s002.docx]

**Epidemiology, histopathologic patterns and clinical characteristics of** **Gestational Trophoblastic Diseases in Ethiopia: A systematic review and meta-analysis study.**

**Abrham Tesfaye Habteyes^1^*****, Fekadeselassie Teferi Mekonen^2^, Ashebir TufaTelila^3^,**

**^1^**Department of Nursing, College of Health Sciences, Ethiopian Defence University, Bishoftu, Ethiopia

**^2^**Department of Pharmacy, College of Health Science, Addis Ababa, Addis Ababa, Ethiopia

**^3^**Department of Midwifery, St’ Paul Hospital Millenium Medical College, Addis Ababa, Ethiopia

^Email Address^

^Abrham T. (^[^abrhamtesfaye95@gmail.com^](mailto:abrhamtesfaye95@gmail.com)^), Ashebir T. (^[^ashebirtufa1616@gmail.com^](mailto:ashebirtufa1616@gmail.com)^) Fekadeselassie T. (^[^fekadeselassie123@gmail.com^](mailto:fekadeselassie123@gmail.com)^),^

^*Corresponding Author^

**Supplementary Appendix**

**Contents**

[List of tables 3](#_Toc199842104)

[List of figures 3](#_Toc199842105)

[JBI critical appraisal checklist for studies reporting prevalence data 4](#_Toc199842106)

[Summary of quality assessment 4](#_Toc199842107)

[Summary of the quality and an agreed level of bias and level of agreement 4](#_Toc199842108)

[Searching strategies 5](#_Toc199842109)

[Regression-based Egger test for small-study effects 5](#_Toc199842110)

[Subgroup analysis Forest plot outputs 6](#_Toc199842111)

[Subgroup analysis by publication year 6](#_Toc199842112)

[Subgroup analysis by region 7](#_Toc199842113)

[Pooled prevalence of different histologic types of GTD in Ethiopia 8](#_Toc199842114)

[Pooled prevalence of complete mole GTD in Ethiopia 8](#_Toc199842115)

[Pooled prevalence of partial mole GTD in Ethiopia 8](#_Toc199842116)

[Pooled prevalence of invasive mole in Ethiopia 9](#_Toc199842117)

[Pooled prevalence of choriocarcinoma in Ethiopia 9](#_Toc199842118)

[Pooled proportion of various clinical features among GTD patients in Ethiopia 10](#_Toc199842119)

[Pooled proportion of vaginal bleeding among GTD patients in Ethiopia 10](#_Toc199842120)

[Pooled proportion of big for date uterus among GTD patients in Ethiopia 10](#_Toc199842121)

[Pooled proportion of passage of vesicles among GTD patients in Ethiopia 11](#_Toc199842122)

[Pooled proportion of vomiting among GTD patients in Ethiopia 11](#_Toc199842123)

[Pooled proportion of hyperthyroidism among GTD patients in Ethiopia 12](#_Toc199842124)

[Pooled proportion of preeclampsia among GTD patients in Ethiopia 12](#_Toc199842125)

[Sensitivity analysis Forest plot outputs 13](#_Toc199842126)

[Pooled prevalence of GTD after trim and fill analysis 13](#_Toc199842127)

# **List of tables**

[**Table 1:** Summary of JBI quality assessment for prevalence of GTD in Ethiopia. 4](#_Toc199842208)

[**Table 2:** Summary of the quality and an agreed level of bias and level of agreement on the methodological qualities of included studies in a meta-analysis. 4](#_Toc199842209)

[**Table 3:** Searching words to study “Epidemiology, histopathology, clinical profiles of gestational trophoblastic diseases in Ethiopia. A systematic review and meta-analysis” 5](#_Toc199842210)

# **List of figures**

[**Figure 1:** Forest plot shows subgroup analysis of pooled prevalence of GTD by publication year in Ethiopia. 6](#_Toc199842217)

[**Figure 2:** Forest plot shows subgroup analysis of pooled prevalence of GTD by region in Ethiopia. 7](#_Toc199842218)

[**Figure 3:** Forest plot shows overall pooled prevalence of complete mole GTD in Ethiopia. 8](#_Toc199842219)

[**Figure 4:** Forest plot shows overall pooled prevalence of partial mole GTD in Ethiopia. 8](#_Toc199842220)

[**Figure 5:** Forest plot shows overall pooled prevalence of invasive mole in Ethiopia. 9](#_Toc199842221)

[**Figure 6:** Forest plot shows overall pooled prevalence of choriocarcinoma in Ethiopia. 9](#_Toc199842222)

[**Figure 7:** Forest plot shows overall pooled proportion of vaginal bleeding among GTD patients. 10](#_Toc199842223)

[**Figure 8:** Forest plot shows overall pooled proportion of big for date uterus among GTD patients in Ethiopia. 10](#_Toc199842224)

[**Figure 9:** Forest plot shows overall pooled proportion of passage of vesicles among GTD patients in Ethiopia. 11](#_Toc199842225)

[**Figure 10:** Forest plot shows overall pooled proportion of vomiting among GTD patients in Ethiopia. 11](#_Toc199842226)

[**Figure 11:** Forest plot shows overall pooled proportion of hyperthyroidism among GTD patients in Ethiopia. 12](#_Toc199842227)

[**Figure 12:** Forest plot shows overall pooled proportion of preeclampsia among GTD patients in Ethiopia. 12](#_Toc199842228)

[**Figure 13:** Sensitivity analysis funnel plot for pooled prevalence of GTD in Ethiopia. 13](#_Toc199842229)

[**Figure 14:** Funnel plot asymmetry shows pooled prevalence of GTD after trim and fills analysis 13](#_Toc199842230)

# **JBI critical appraisal checklist for studies reporting prevalence data**

## **Summary of quality assessment**

**Table 1:** Summary of JBI quality assessment for prevalence of GTD in Ethiopia.

| **List of studies** | **List of questions** | | | | | | | | | | | | | | | | | | **Total Score** | |
| --- | --- | --- | --- | --- | --- | --- | --- | --- | --- | --- | --- | --- | --- | --- | --- | --- | --- | --- | --- | --- |
|  | **Q1** | | **Q2** | | **Q3** | | **Q4** | | **Q5** | | **Q6** | | **Q7** | | **Q8** | | **Q9** | |  |  |
|  | R1 | R2 | R1 | R2 | R1 | R2 | R1 | R  2 | R1 | R2 | R1 | R2 | R1 | R  2 | R1 | R2 | R1 | R2 | **R1** | **R2** |
| Nigussie D. et al. | Y | Y | N | Y | Y | Y | Y | Y | Y | Y | Y | Y | Y | Y | Y | Y | Y | U | **6** | **8** |
| Yesuf A. et al. | Y | Y | Y | Y | Y | Y | Y | Y | Y | Y | Y | Y | Y | Y | Y | Y | Y | Y | **9** | **9** |
| Yilma M. et al. | Y | Y | Y | Y | Y | Y | Y | Y | Y | Y | Y | Y | Y | N | U | Y | Y | Y | **8** | **8** |
| Hayelom K. et al. | Y | Y | Y | Y | U | Y | Y | Y | Y | Y | Y | Y | N | Y | Y | Y | Y | Y | **7** | **9** |
| Yibrah B. et al. | Y | Y | Y | Y | Y | Y | Y | Y | U | N | Y | Y | Y | Y | Y | Y | Y | Y | **8** | **8** |
| Alemnew S. et al. | N | U | U | Y | Y | N | U | Y | Y | Y | Y | Y | U | Y | N | Y | Y | Y | **4** | **6** |
| Ahmed B. et al. | Y | N | Y | Y | Y | Y | Y | U | Y | Y | Y | N | Y | Y | Y | N | Y | Y | **9** | **5** |
| **R1:** reviewer 1(AT), **R2:** reviewer 2(FT) | | | | | | | | | | | | | | | | | | | | |

# **Summary of the quality and an agreed level of bias and level of agreement**

**Table 2:** Summary of the quality and an agreed level of bias and level of agreement on the methodological qualities of included studies in a meta-analysis.

| **Study** | **Overall agreement and precision** | | | **JBI quality score (from a total 1 to 9-point score)** |
| --- | --- | --- | --- | --- |
|  | **Percentage of agreement** | **Kappa value** | **Level of agreement** |  |
| Negussie D. et al. | 71% | 0.74 | Substantial | 7 |
| Yesuf A. et al. | 100% | 1 | Perfect | 9 |
| Yilma M. et al. | 100% | 1 | Perfect | 8 |
| Hayelom K. et al. | 86% | 0.78 | Substantial | 8 |
| Yibrah B. et al. | 100% | 1 | Perfect | 8 |
| Alemnew S. et al. | 86% | 0.78 | Substantial | 5 |
| Ahmed B. et al. | 65% | 0.67 | Substantial | 7 |
| **NB: JBI:** Joanna Briggs Institute | | | | |

# **Searching strategies**

**Table 3:** Searching words to study “Epidemiology, histopathology, clinical profiles of gestational trophoblastic diseases in Ethiopia. A systematic review and meta-analysis”

| **Condition** | (Magnitude) OR (Prevalence) OR (Burden) |
| --- | --- |
|  | **AND** |
|  | (Clinical profiles) OR (Profiles) OR (Clinical features) OR (Clinical types) OR (Clinical manifestations) |
|  | **AND** |
|  | Histopathology |
| **AND** | |
| **Context** | Ethiopia |
| **AND** | |
| **Population** | (Women with Gestational Trophoblastic Diseases) OR (Gestational Trophoblastic Diseases) OR (Hydatidiform mole) OR (Molar pregnancy) OR (Choriocarcinoma) OR (Placental-site trophoblastic tumor) OR (Epithelioid trophoblastic tumor) OR (Gestational Trophoblastic Neoplasia) OR (Invasive moles) OR (GTD) OR (GTN) |

# **Regression-based Egger test for small-study effects**


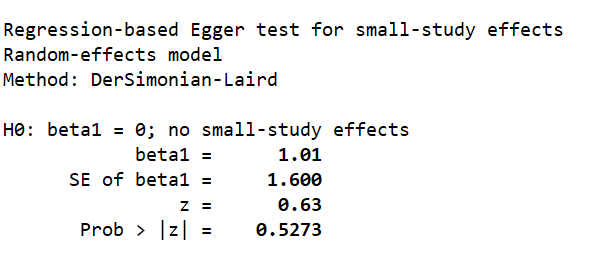


# **Subgroup analysis Forest plot outputs**

## **Subgroup analysis by publication year**

**Figure 1:** Forest plot shows subgroup analysis of pooled prevalence of GTD by publication year in Ethiopia.

## **Subgroup analysis by region**

**Figure 2:** Forest plot shows subgroup analysis of pooled prevalence of GTD by region in Ethiopia.

# **Pooled prevalence of different histologic types of GTD in Ethiopia**

## **Pooled prevalence of complete mole GTD in Ethiopia**

 **Figure 3:** Forest plot shows overall pooled prevalence of complete mole GTD in Ethiopia.

## **Pooled prevalence of partial mole GTD in Ethiopia**

**Figure 4:** Forest plot shows overall pooled prevalence of partial mole GTD in Ethiopia.

## **Pooled prevalence of invasive mole in Ethiopia**

**Figure 5:** Forest plot shows overall pooled prevalence of invasive mole in Ethiopia.

## **Pooled prevalence of choriocarcinoma in Ethiopia**

**Figure 6:** Forest plot shows overall pooled prevalence of choriocarcinoma in Ethiopia.

# **Pooled proportion of various clinical features among GTD patients in Ethiopia**

## **Pooled proportion of vaginal bleeding among GTD patients in Ethiopia**

**Figure 7:** Forest plot shows overall pooled proportion of vaginal bleeding among GTD patients.

## **Pooled proportion of big for date uterus among GTD patients in Ethiopia**

**Figure 8:** Forest plot shows overall pooled proportion of big for date uterus among GTD patients in Ethiopia.

## **Pooled proportion of passage of vesicles among GTD patients in Ethiopia**

**Figure 9:** Forest plot shows overall pooled proportion of passage of vesicles among GTD patients in Ethiopia.

## **Pooled proportion of vomiting** **among GTD patients in Ethiopia**

**Figure 10:** Forest plot shows overall pooled proportion of vomiting among GTD patients in Ethiopia.

## **Pooled proportion of hyperthyroidism among GTD patients in Ethiopia**

**Figure 11:** Forest plot shows overall pooled proportion of hyperthyroidism among GTD patients in Ethiopia.

## **Pooled proportion of** **preeclampsia among GTD patients in Ethiopia**

**Figure 12:** Forest plot shows overall pooled proportion of preeclampsia among GTD patients in Ethiopia.

# **Sensitivity analysis Forest plot outputs**

**Figure 13:** Sensitivity analysis funnel plot for pooled prevalence of GTD in Ethiopia.

# **Pooled prevalence of GTD after trim and fill analysis**

**Figure 14:** Funnel plot asymmetry shows pooled prevalence of GTD after trim and fills analysis
